# Bronchoscopic Management of Endobronchial Atypical Carcinoid With Argon Plasma Coagulation and Laser: A Rare Case With Literature Review

**DOI:** 10.7759/cureus.13862

**Published:** 2021-03-12

**Authors:** Andrew Talon, Melinda Wang, Ali Saeed

**Affiliations:** 1 Internal Medicine, Creighton University School of Medicine, St. Joseph's Hospital and Medical Center, Phoenix, USA; 2 Interventional Pulmonology, Norton Thoracic Institute, St. Joseph's Hospital and Medical Center, Phoenix, USA

**Keywords:** atypical carcinoid, endobronchial tumor, interventional pulmonology, bronchocele

## Abstract

Atypical carcinoid belongs to a spectrum of neuroendocrine tumors that can present as central airway obstruction. We treated a 58-year-old female who presented with recurrent pneumonia. Flexible bronchoscopy showed complete obstruction of the tumor in the right lower lobe. The tumor was excised by electrocautery snare followed by laser and argon plasma coagulation (APC). Endobronchial biopsy showed atypical carcinoid with lymph node metastasis. Succeeding bronchoscopic management, the patient’s symptoms improved. In our patient, bronchoscopy with laser and APC was performed to prevent tumor recurrence after resection and reduce the risk of recurrent postobstructive pneumonia. Surveillance computed tomography at six months showed no evidence of recurrence. Bronchoscopic management should be considered in poor surgical candidates or patients with metastatic disease.

## Introduction

Atypical carcinoid (AC) is a rare neuroendocrine tumor that can present as an obstructing endobronchial mass [1]. Contrasted to typical carcinoid, ACs have a higher malignant potential with lower survival rates [2,3]. Localized tumors have been historically managed by surgical resection as the mainstay of treatment [2,3]. Recent literature however has shown excellent long-term outcome after first-line bronchoscopic therapy of endobronchial carcinoid tumors in a subgroup of patients [2-4]. Compared to surgical resection, bronchoscopic management is minimally invasive and parenchyma sparing [2,3]. We present a case of endobronchial AC managed with bronchoscopy.

## Case presentation

**Figure 1:**
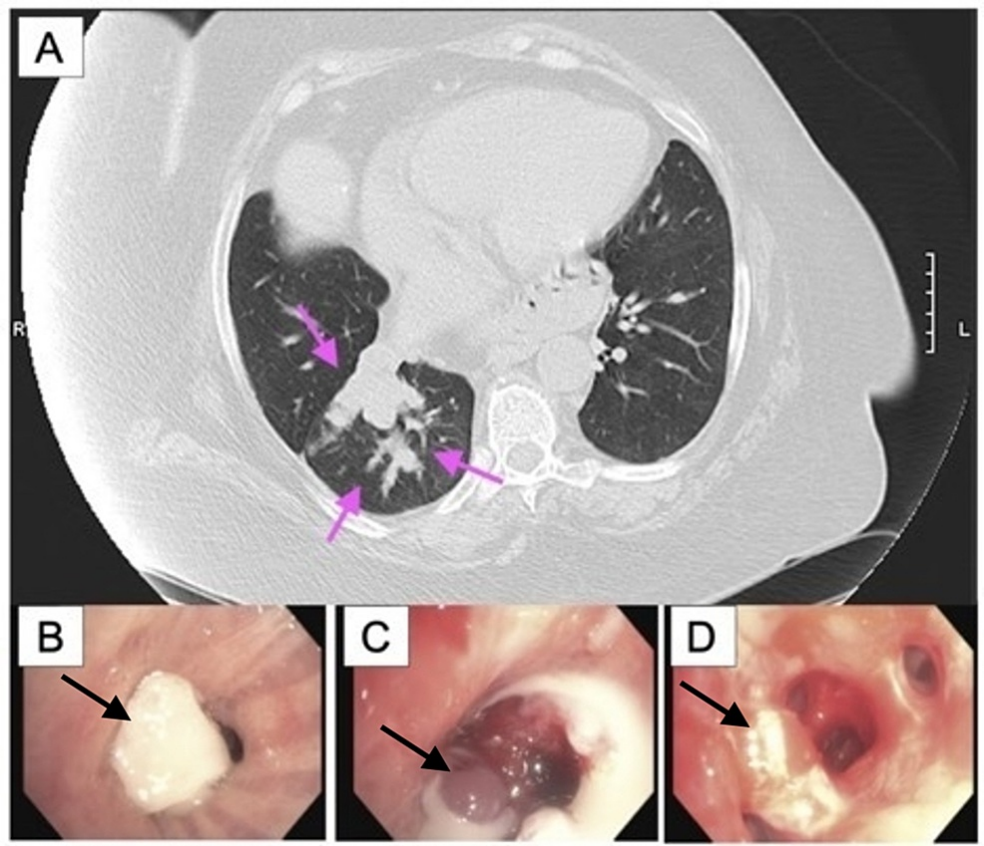
(A) CT showing an RLL consolidation with projecting opacities characteristic of a bronchocele. (B) Bronchoscopy showing proximal obstruction of the tumor at the level of the RLL by a bronchocele. (C) Evacuation of the mucous plug and pus formation with cryoprobe and suction revealing an obstructing endobronchial tumor. (D) An atypical carcinoid tumor with origin in the RLL medial base region following debulking with visualization of distal airways. RLL - right lower lobe

**Figure 2:**
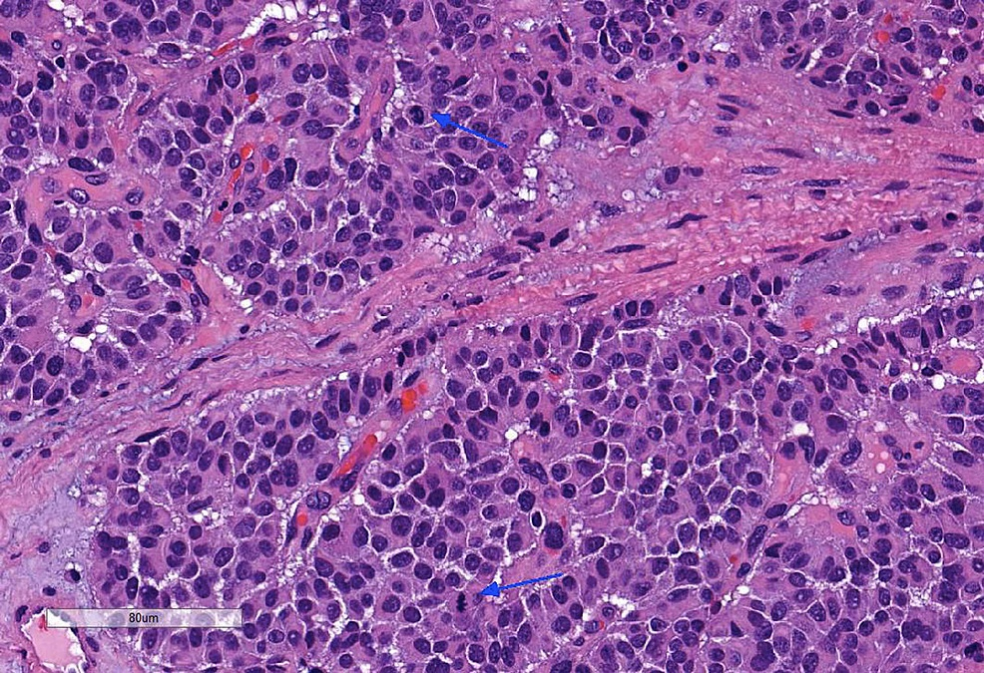
Histolopathology of RLL endobronchial tumor. The presence of necrosis and increased mitotic count (blue arrows) are features consistent with atypical carcinoid.

**Figure 3:**
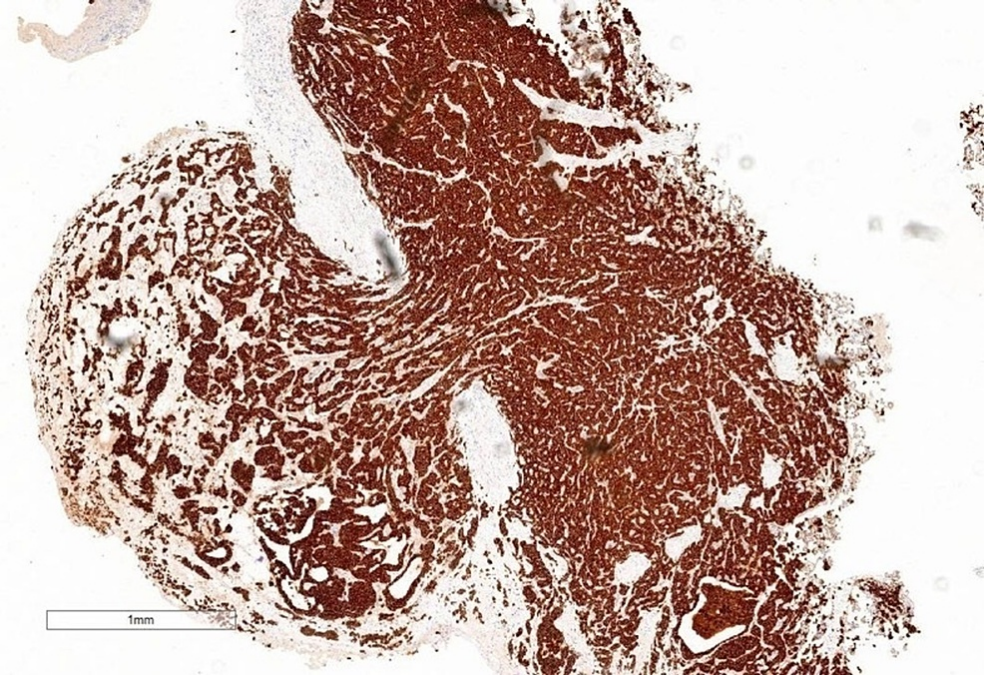
Immunohistochemical stains. The tumor was strongly positive for CD56 and synaptophysin. The staining pattern was consistent with atypical carcinoid tumor primary to the lung.

A 58-year-old female developed dyspnea and a productive cough. She was diagnosed with pneumonia and received antibiotics. Despite this, her symptoms worsened. Computed tomography (CT) revealed lobulated finger-like opacities with consolidation in the right lower lobe (RLL) (Figure 1A). Flexible bronchoscopy found that the RLL was completely obstructed (Figure 1B). Removal of the mucoid impaction revealed an endobronchial tumor originating from the RLL medial basal segment (Figure 1C). The tumor was then excised piecemeal using an electrocautery snare and cryoprobe (Video 1). Tumor base coagulation was performed using diode laser and argon plasma coagulation (APC) for hemostasis (Video 1). All RLL branches were ultimately visualized following debulking (Figure 1D). The mediastinal staging was then performed with endobronchial ultrasound. Fine needle aspiration biopsy confirmed a neuroendocrine neoplasm favoring primary pulmonary AC with subcarinal and right lower paratracheal lymph node metastasis (Figures 2, 3). The patient was presented to the tumor board, deciding that she was a poor surgical candidate. Following our bronchoscopic intervention, the patient’s cough and dyspnea were immediately improved. She was referred to oncology and was treated with carboplatin-paclitaxel chemotherapy with concurrent fractionated radiotherapy. Surveillance CT at six months showed no evidence of recurrence.

**Video 1:** Dr. Ali Saeed, MD, Medical Director of Interventional Pulmonology (Norton Thoracic Institute, Phoenix, AZ) performing flexible bronchoscopy in a patient with an obstructing right lower lobe endobronchial mass and bronchocele. Tumor was excised with electrocautery snare and cryoprobe followed by diode laser and APC to prevent tumor recurrence. APC - argon plasma coagulation

 

 

## Discussion

ACs belong to a spectrum of neuroendocrine tumors that account for 2% of all primary lung tumors [1]. ACs account for 10% of this subgroup, while the remaining are typical carcinoid, large cell carcinoma and small cell carcinoma [3,4]. The literature on endobronchial management of AC tumors largely comes from surgical series utilizing bronchoscopic resection as an adjunct with surgery [2,5]. A recent meta-analysis by Reuling et al. noted that no randomized trials to compare bronchoscopic management alone versus surgical treatment of pulmonary carcinoid tumors have been performed at present [6]. The obvious concern is the potential for tumor regrowth despite endobronchial treatment. Another concern is the development of the late recurrent disease that would be beyond curative surgery [3]. Conversely, a prospective study noted a 7.8% recurrence in the initial bronchoscopic treatment group requiring surgery without an uncompromised outcome in survival [3].

Clinically, it is difficult to discern whether there is a remnant tumor at the resection margin during the endobronchial intervention. Previous studies have reported that additional laser ablation to the tumor bed has been efficacious to prevent tumor recurrence [7-9]. In our case, laser and APC therapy was performed after resection. Our technique also reduces the risk of recurrent postobstructive pneumonia. Snare electrocautery and cryoprobe following debulking were preferred due to low risk for hemorrhage.

Not all patients with AC can be treated by bronchoscopy because many tumors also include extraluminal components also known as the "iceberg phenomenon." A recent study evaluating the potential prognostic factors when considering primary bronchoscopic management found that small intraluminal tumors of ≤2 cm without signs of extraluminal growth were the most suitable for endobronchial resection, while all other tumors larger than 2 cm should be referred to surgery [3,6]. Currently, there are no guidelines for preoperative mediastinal lymph node staging for endobronchial carcinoid tumors [3-7]. Previous studies have shown that endobronchial intervention, without lymph node dissection, did not influence survival in patients [3,4].

Central carcinoid tumors may be associated with a mucoid impaction [10]. A bronchocele, or mucoid impaction, is a common radiographic finding best characterized as tubular opacities, also known as the finger-in-glove sign [11]. Bronchoceles are most associated with benign neoplastic processes including lipomas, endobronchial hamartomas, and papillomatosis [11]. Rarely are they associated with malignancies [11].

## Conclusions

AC tumors with lymph node involvement can be effectively managed with bronchoscopic resection in carefully selected patients who are poor surgical candidates. ACs can also be associated with a bronchocele, so we recommend bronchoscopy for direct visualization and to rule out malignancy. Given the rarity of this tumor, patients should be presented to the tumor board as a multidisciplinary approach is favored. Further studies comparing endobronchial tumor ablation techniques seem warranted. 
